# Manufacturing Technology of Composite Materials—Principles of Modification of Polymer Composite Materials Technology Based on Polytetrafluoroethylene

**DOI:** 10.3390/ma10040377

**Published:** 2017-03-31

**Authors:** Anton Panda, Kostiantyn Dyadyura, Jan Valíček, Marta Harničárová, Jozef Zajac, Vladimír Modrák, Iveta Pandová, Peter Vrábel, Ema Nováková-Marcinčínová, Zdeněk Pavelek

**Affiliations:** 1 Faculty of Manufacturing Technologies with a seat in Prešov, Technical University of Kosice, Bayerova 1, 080 01 Prešov, Slovakia; jozef.zajac@tuke.sk (J.Z.); vladimir.modrak@tuke.sk (V.M.); iveta.pandova@tuke.sk (I.P.); peter.vrabel.2@tuke.sk (P.V.); novakmarcincin@me.com (E.N.-M.); 2 Department of Applied Materials Science and Technology of Constructional Materials, Faculty of Technical Systems and Energy Efficient Technologies, Sumy State University, 40007 Sumy, Ukraine; dyadyura@pmtkm.sumdu.edu.ua; 3 Institute of Physics, Faculty of Mining and Geology, VŠB—Technical University of Ostrava, 17. Listopadu 15/2172, 708 33 Ostrava-Poruba, Czech Republic; jan.valicek@vsb.cz (J.V.); marta.harnicarova@vsb.cz (M.H.); 4 RMTVC, Faculty of Metallurgy and Materials Engineering, VŠB—Technical University of Ostrava, 17. Listopadu 15/2172, 708 33 Ostrava-Poruba, Czech Republic; 5 OKD, HBZS, a.s., Ostrava—Radvanice 716 00, Czech Republic; pavelek@hbzs-ov.cz

**Keywords:** polytetrafluoroethylene, polymer composites materials, fillers, modification of the polymer matrix, adhesion, friction, manufacturing technology mechanical properties

## Abstract

The results of the investigations into the technological formation of new wear-resistant polymer composites based on polytetrafluoroethylene (PTFE) filled with disperse synthetic and natural compounds are presented. The efficiency of using PTFE composites reinforced with carbon fibers depends on many factors, which influence the significant improvement of physicomechanical characteristics. The results of this research allow stating that interfacial and surface phenomena of the polymer–solid interface and composition play a decisive role in PTFE composites properties. Fillers hinder the relative movement of the PTFE molecules past one another and, in this way, reduce creep or deformation of the parts, reducing the wear rate of parts used in dynamic applications as well as the coefficient of thermal expansion. The necessary structural parameters of such polymer composites are provided by regimes of process equipment.

## 1. Introduction

The materials for sealing elements have special requirements, especially considering the conditions of operation of such equipment: long production cycle without service, reverse of natural movement, lack of special lubricants, influence of hostile environment and increased temperatures. The widespread use of polytetrafluoroethylene composites in friction units and seals of various kinds of machinery and equipment is due to features of the molecular and supramolecular structure of polytetrafluoroethylene (PTFE), which ensure the implementation of a unique combination of deformation and strength, tribotechnical, anticorrosive, thermophysical, and other service characteristics, and determine the efficient use of these products [1]. For decades, fillers have been successfully used to reduce the wear of PTFE. The wear-reducing mechanism of fillers in PTFE-based composites remains a topic of debate. Various fiber and particle fillers have successfully reduced the wear of PTFE by several orders of magnitude, but they also increase friction coefficients and abrade favorable transfer films and the counterface, both of which limit the effectiveness of the filler. Additionally, the high filler loadings (~20%) needed for significant wear reductions have detrimental effects on the beneficial frictional, thermal and chemical properties that make PTFE so attractive to designers. The use of nanoparticles has the potential to eliminate many of the limitations of traditional fillers in a PTFE matrix. Low loadings of nanoparticles have imparted impressive improvements in mechanical properties such as strength, Young’s modulus and strain to failure to other polymeric matrices. They have also been found to reduce abrasion and promote transfer film development. Despite the success of microfillers in abating severe wear of PTFE and the demonstrated benefits of nanoparticles on the properties of other polymer matrices, there was a sentiment in the field that nanoscopic fillers were ineffective in reducing the wear of PTFE. This is understandable if we take into consideration the complexity of interfacial interactions in multicomponent polymer systems and their connection with properties of polymer composites. The further production development of polymer composite materials (PCM) based on PTFE should follow the scientific principles of polymer and composites technology, applied materials science and physicochemical mechanics of composite structures [2,3,4,5,6,7,8]. The research of preparing a matrix and fillers and the development of PTFE-based composites solve an important scientific and technical problem and determine the relevance of this paper.

The combination of the theoretical research of structural and phase transformations occurring in filling the PTFE matrix of a composite with fillers and experimental research allows formulating a scientifically based approach to the forecasting and targeted regulation of PTFE composite properties [9,10,11,12].

The aim of this paper is to generalize the research results of factors of the technological preparation process of components influencing physical, mechanical, and performance properties of composite materials and determine their optimum values on the basis of deepening scientific understanding of the structure formation processes of composites with hybrid fillers. 

The studying of physicomechanical and tribotechnical characteristics allows controlling the properties of PTFE composites, which is an important problem in modern polymer materials science, and provide the consumers with composite materials of forecasted necessary properties, up to the world’s best analogs.

## 2. Materials and Methods of Research

### 2.1. Research Materials

PTFE is made of a carbon backbone chain, and each carbon has two fluorine atoms attached to it. Its chemical formula is (–CF2–CF2–)_n_. PTFE is a completely fluorinated polymer produced when the monomer tetrafluoroethylene (TFE) undergoes free radical vinyl polymerization [1,6,10,11,12].

PTFE is an amorphous-crystalline polymer with the melting point of the crystallites of +327 °C and the vitrification temperature of the amorphous phase −120 °C; it has a high degree of crystallinity; and a large number of crystallites is observed even after quenching (rapid cooling starting from melting point) [8,13]. The degree of crystallinity of PTFE depends on cooling speed in the case of the thermal treatment (sintering) of pressed materials in the range of temperatures 300–370 °C. The maximum content of crystalline phase is observed at the minimum speed of cooling when favorable conditions for forming crystallites [8] are created. The resulting polymer is a loose, fibrous and cloggy white powder [10,13]. 

ISO 13000-1:2005 [14] specifies the requirements for processed unfilled PTFE products which may occur in several forms. The PTFE used to make the semi-finished product is described in ISO 12086-1:2006 [15] and, as provided in this standard, may contain up to 1% co-monomer. The PTFE used to make the semi-finished product may be virgin, reprocessed or recycled resin. The addition of up to 1.5% (by mass) pigment or colorant is permitted. This part of ISO 13000-1:2005 [14] allows for four grades based on tensile strength and elongation at break. The semi-finished products can be as-processed (type P) or dimensionally stabilized (type S) and may also have specified electrical properties or other properties when they are required for an application.

The basic physicomechanical and tribotechnical characteristics of the material based on PTFE are listed in Table 1.

The parameters of characteristics of mechanical properties of PTFE are considerably determined by the degree of crystallinity, i.e., the content of crystalline phase in the structure of polymer [13]. In modern materials science, regarding PTFE-based composites for engineering appointment, there are no common methodological approaches for obtaining commercial batches of materials with stable parameters of strength and wear resistance within one brand. It is experimentally established that there is a parameter spread of service characteristics of composites of one brand because of the lack of reasonable criteria defining quality of initial components, i.e. PTFE and the carbon fibers.

However, due to the specific of structural and morphological characteristics of macromolecules and disperse particles of PTFE and CF, potential opportunities of PTFE-based composites components are not realized fully. As stated earlier, the urgent task is to develop structures and technology of PTFE-based composites with increased parameters of strength and tribotechnical characteristics by means of directional control of physicochemical and thermal processes at the interface between the matrix and the carbon filling agent.

Hydrated cellulose carbon fibers (CF) UTM-8-1s (product model [16]) obtained by chemical processing in an aqueous solution of fire-retardants Na_2_B_4_O_7_·10H_2_O + (NH_4_)_2_HPO_4_ and annealing at temperatures of 723 ± 20 K (450 ± 20 °C) in a natural gas (CH_4_) environment was used as the main fiberfill.

Its chemical composition and properties are shown in Table 2 and Table 3, respectively.

The increase in the tensile elongation at break is significant, particularly since elongation is inversely proportional to brittleness [17,18].

### 2.2. Methods of Research

The methodology of studying the composite properties included determining the density ρ (g/cm^3^), breaking strength σ_b_ (MPa), relative elongation δ (%), and wear intensity I·10^−7^ (mm^3^/N·m) (volume lost per unit normal load per distance of sliding) in accordance with the regulations.

Test samples were obtained by cold molding technology (molding pressure P_mold_ = 50.0–70.0 MPa), followed by the free sintering of tablet blanks in air at 365 ± 5 °C at a speed of heating–cooling of 40 °C/h.

The tests of strength and relative elongation at break were performed on ring samples of 50 × 40 mm in diameter and 10 mm in height using rigid half-discs (ISO 527-1:2012 [19] and standard ASTM D638-14 [20]) on an R-1 disruptive installation (ASTM D695-15 [21]) at the motion speed of sliding member of 0.25 cm/min.

The density ρ (g/cm^3^) of the samples was determined by hydrostatic weighing (ASTM D1505-10 [22]).

The study of the wear rate was carried out on an SMT-1 serial friction machine according to the “partial insertion-shaft” scheme.

The set of samples was tested in the friction mode without external lubrication at the sliding speed of V = 1 m/s and pressure of P = 1 MPa. The counterbody was a roller having a diameter of 48 mm made of steel 45 (Rockwell hardness number HRC 25, Ra = 0.38 micron). The partial insertion was manufactured using PTFE and was a sector with the width of 16 mm from the ring—diameter of 80 on a diameter of 60 mm—and the height of 9 mm.

The rate of mass wear was assessed by the mass loss of samples per unit time. The magnitude of wear of the samples was determined gravimetrically on an analytical balance within the accuracy of 10^−5^ grams and transferred to the intensity of wear. The moment of friction was recorded using a Termodat 17E3 (Sistemy kontrolya, Perm, Russia) device.

In the assessment of the intensity of wear, the mean square error was regulated by measurement errors of the mass of a sample and the speed and duration of friction, and did not exceed 5%.

The study of the supramolecular structure of activated PTFE powder was carried out using a TESCAN MIRA 3 LMU scanning electron microscope of high resolution (TESCAN, Brno, Czech Republic).

The surface morphology of CF was carried out using a raster electron microscope (REM-200) (Selmi, Sumy, Ukraine)

The processing of experimental data was performed using the mathematical design of experiments and mathematical statistics.

## 3. Methods of PTFE-MATRIX Modification

The review of literature and patents [23,24,25,26,27,28,29,30,31,32,33,34] reveals a low potential of traditional technological approaches in obtaining PTFE-based PCM. However, various advanced technological methods that allow obtaining composite materials and products with the required performance properties may be implemented. They are the synthesis of polymer matrices of different compositions and structures [35,36]; composition of polymer and oligomer mixture with different levels of interaction [37]; modification of matrices of various origins by aimed restructuring and structural interaction energy impact [38]; matrix modifying by additions of activators of different size, shape and properties [39,40,41,42]; and formation of nanophase matrices with significantly different characteristics [43,44].

**Figure 1:**
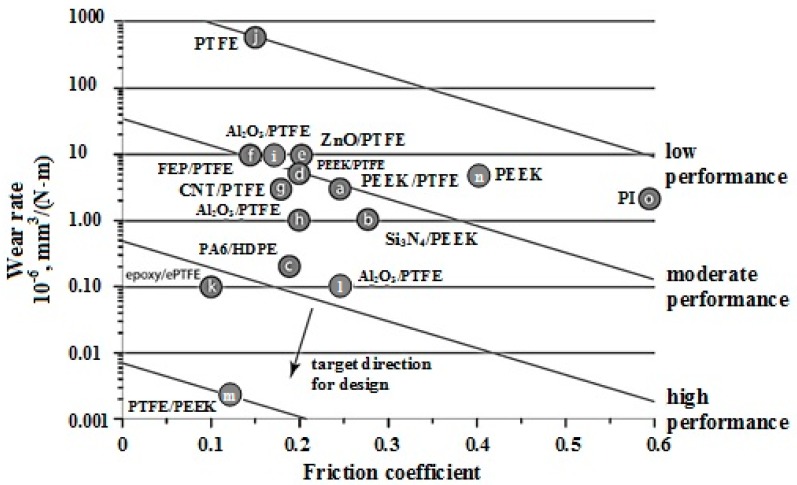
Wear rate plotted versus friction coefficient for various solid lubricating polymeric composites, unfilled polymers, and polymer blends. The target region is the lower left hand corner, a region of ultra low wear rate and friction coefficient. The data points are labeled with the constituents and listed as: (a) PTFE/PEEK (polyether-ether-ketone) composite [45]; (b) Si_3_N_4_/PEEK nanocomposite [46]; (c) Polyamide 6 (PA6)/HDPE (high density polyethylene) blend [47]; (d) PTFE/PEEK composite [48]; (e) ZnO/PTFE nanocomposite [49]; (f) Fluorinated ethylene propylene/polytetrafluoroethylene (FEP/PTFE) composite [50]; (g) Carbon nanotube/polytetrafluoroethylene (CNT/PTFE) nanocomposite [51]; (h) Al_2_O_3_/PTFE nanocomposite [52]; (i) Al_2_O_3_/PTFE nanocomposite and (j) unfilled PTFE [53]; (k) epoxy/ePTFE (expanded polytetrauoroethylene) composite [54]; (l) Al_2_O_3_/PTFE nanocomposite [55]; (m) PEEK/PTFE composite and (n) unfilled PEEK [55]; and (o) unfilled polyimide (PI) unpublished, V = 50.8 mm/s, P = 6.25 MPa, reciprocating pin-on-disk tribometer.

Figure 1 shows the wear rate plotted versus friction coefficient for various unfilled polymers, polymer blends, and polymer composites used in tribology studies [8,9,10,11,12,13,23,24,25,26,27,28]. While tribological performance does not have a single unique definition, broadly speaking, solid lubricants with low wear rates and low friction coefficients are desirable. For practical purposes, a designer might include constant performance guidelines (Figure 1 illustrates how such guidelines might be used) whose slopes depend on the relative importance of friction coefficient and wear rate for a specific application (note: wear rates are on a log scale).

**Figure 2:**
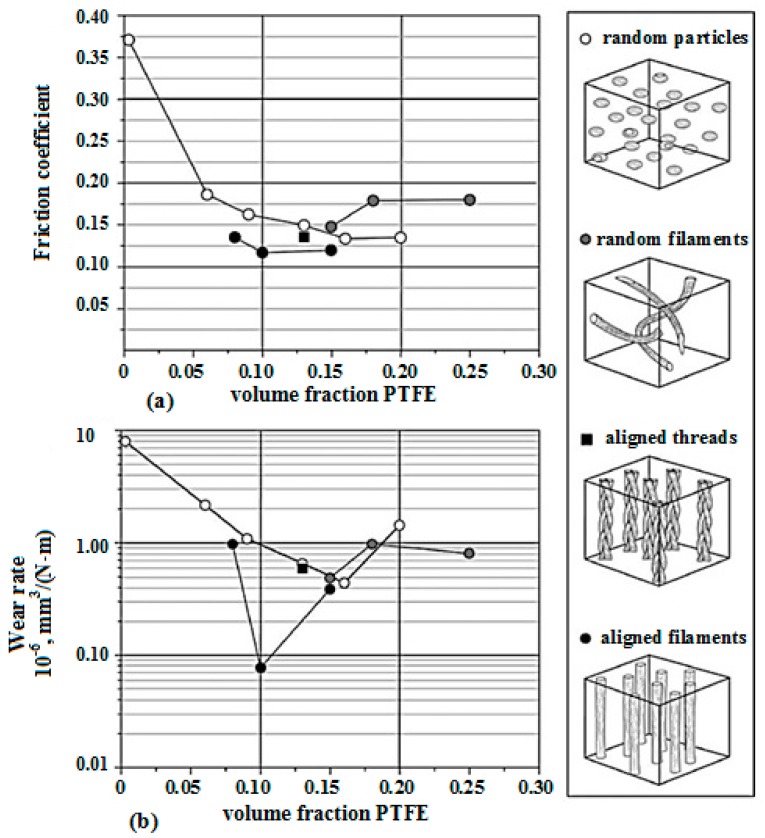
(**a**) Friction coefficient versus PTFE filler loading. At low loadings of aligned ePTFE filaments, the friction coefficient was slightly less than that of the powder filled composites at similar loadings. (**b**) Steady state wear rates plotted against PTFE filler loading. Composites with expanded PTFE filaments aligned normal to the sliding direction demonstrated the best wear rates; the performance of randomly oriented filaments was similar to the powder PTFE filled PEEK composites [3].

There are significant efforts dedicated to the research and development of low friction, low-wear solid lubricants with traditional particle and fiber fillers, many of which have successfully transferred to application. In this particular composite, a soft PTFE film is preferentially drawn from the composite to separate the surfaces, protecting the relatively soft polymeric material from direct asperity contact, and providing a low shear friction reducing film to accommodate the sliding motion. This is called a transfer film. Friction coefficients for unfilled PEEK were relatively high and noisy with average values of μ = 0.37. The addition of PTFE reduced the friction coefficient for all loading conditions. These data are plotted against filler loading in Figure 2. The addition of 8 % (v/v) aligned ePTFE filaments reduced this friction coefficient to an average of μ = 0.13 and 10 % (v/v) aligned filaments yielded a friction coefficient of μ = 0.11.

## 4. Industrial Technology for PCM with PTFE-MATRIX

Filled polymer technology is the process of mixing the initial components. It determines the ultimate macro- and microstructure of composite materials, and their physicomechanical and tribotechnical characteristics. The physicomechanical and tribotechnical characteristics of filled polymers relate not only to the interaction between components, but also to the change in structure and properties of PCM associated with phase and structural transformations.

The technological process of obtaining composite PTFE-based materials has been changing with difficulty [56,57].

During the process of sintering, a pressed material at the stage of the fusion of PTFE particles and fillers, the chemical, physical and mechanical performance properties of the future product are developed, so the sintering and the subsequent heat treatment is an important step in the process of manufacturing products from a composite. 

In practical application, it is not always possible to achieve the best production modes of obtaining PTFE composites. The solution is seen in the maximum intensification of each stage of the process.

The structural links of this chain may be supplemented by new elements (processes) [58,59,60] or vice versa: some parts of the links may be excluded from the process as unnecessary. The comprehensive approach to each stage of the PTFE composite making will identify the most important factors of energy fields’ impact on the structure-forming activity of the composition ingredients and the identification of the criteria of their compatibility. At the same time, the technological modes of gradual impact on the composition (drying, milling, mixing, pressing, and heat treatment) play an important role as well as the ordering of structure elements of the composition and activation of physical and chemical interactions at the phase boundary.

## 5. Preparation of PTFE-MATRIX by Mechanical Activation

The activation of PTFE powder was carried out on an МRP-1М (Public Joint Stock Company “Sumy Machine-Building Science-and-Production Association”, Sumy, Ukraine) mill with a varying frequency of working parts rotation in the interval *n* = 5000–9000 min^−1^ and during the experimentally determined time interval τ = 3–8 min.

The morphology and fractional composition of PTFE under mechanical activation with a number of rotations lower than 5000 min^−1^ does not provide any equal distribution of the activated PTFE particles in the material, and with a number of rotations higher than 9000 min^−1^ it leads to the coagulation of the activated particles and the formation of a heterogeneous material structure.

The activated powder was obtained by dry milling in a high speed blade mixer МRP-1М. The difference between the structures of activated and non-activated PTFE leads to differences in physical and mechanical properties (Table 4).

The mechanochemical destruction of PTFE macromolecules occurs during the previous activation with the formation of radical fragments [5,17,18,25]. The presence of the active surface of the filler particle, on the one hand, and the PTFE macromolecule free radical, on the other hand, may initiate the polymer grafting reaction with the filler [26]. Although such reactions, forming chemical bonds between the polymer and the filler surface, take place only in active centers and have a likely nature, their contribution to strengthening the composite material is very significant [18,25].

This also leads to the preservation of extra energy by the polymer substance, to the changes of the thermodynamic properties, and the increase of its reactivity.

Furthermore, the mechanical load as a result of the particles collision leads to metastable states of surface layers of polymer particles. These particle collisions progress for several seconds, and are followed by the local increase of temperature and pressure at the contact surfaces points [23,24]. All these phenomena lead to the formation of uncompensated valences on the surface, promote interactions between the filler particles of composite, and initiate the polymerization reaction of monomers or the formation of chemical bonds with polymer radicals [12,31,33,44].

It is determined that the mechanical activation mode of PTFE matrix with the rotation numbers of mill working parts *n* = 9000 min^−1^ for 5 min is the best achieved result. In addition to the above, the breaking strength σ_b_ = 24.8 MPa, relative elongation δ = 415%, wear intensity I = 61.0 × 10^−7^ mm^3^/N·m, whereas the non-activated PTFE has σ_b_ = 9.5 MPa, δ = 96%, I = 11.33 × 10^−7^ mm^3^/N·m.

The energy impact on unfilled PTFE increases the parameters of its deformation and strength characteristics (breaking strength by 2.6 times, and breaking elongation by 4.3 times) while maintaining high tribotechnical indices. This is obviously due to the formation of new reaction centers, and the increase of the individual fragments and macromolecules surface energy as a result of elastic and plastic deformations.

The increase of PTFE wear resistance in the mechanical activation is associated with the decrease in the degree of crystallinity and the increase in the average interlayer distance during the frictional interaction and the structural adjustability of the modified PTFE under conditions of friction and demonstration of synergistic effects of self-organization tribostructures with high wear resistance [38].

**Figure 3:**
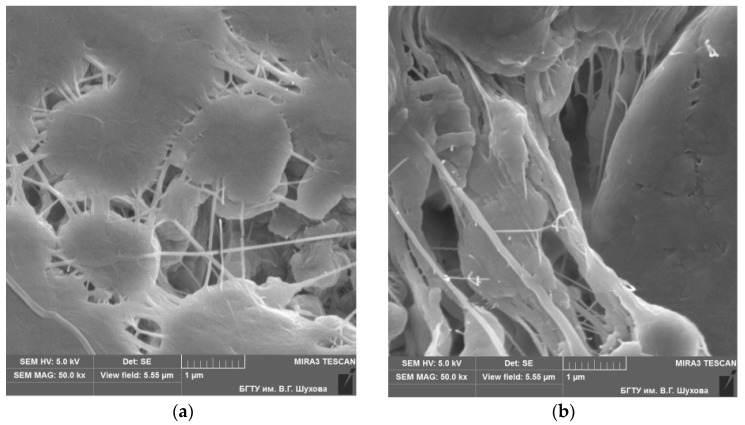
Changes of PTFE supramolecular structure. Structure of PTFE before (**a**) and after (**b**) mechanical activation (×50,000). Scale bar: 1 µm.

Using the methods of electron microscopy, it was established that the PTFE supramolecular structure under the mechanical activation has been significantly changed from a disordered lamellar structure to a spherulitic structure with higher ordering (Figure 3).

In the structure of PTFE samples after the mechanical activation, strands of fibers with a length of 10 to 50 microns and a diameter from 10 to 100 nm (Figure 3b) are observed, which are absent in the non-activated PTFE structure (Figure 3a).

The difference between the particle morphology of fractions is because the products obtained at different thermobaric effects have different ratios of molecular components, and each of them is intended to the construction of certain morphological formations.

Thus, due to the high physicomechanical and wear resistance indices, mechanically activated PTFE and its compositions may be used for manufacturing antifriction parts of machinery and equipment moving joints.

## 6. Preparation and Modification Technology of Fibrous Filler

The surface of carbon fiber (CF) is inert under usual conditions [5]. The PTFE composition with CF is a complex heterogeneous system with numerous surfaces of phase distribution.

Physicochemical processes in boundary layers during forming the composition make a significant contribution to the structure formation and, thus, to major operating properties in such compositions.

The formation of reaction surfaces of PTFE matrix and CF is an important scientific and practical task and solving this guarantees obtaining and reproducing planned properties of the composite.

It was suggested to modify the surface of carbon fiber by different methods in order to provide the technological combining of carbon fiber with polymer matrix in PTFE-based antifriction composite materials [57,59,60]. Such filler processing allows increasing composite materials characteristics, which depend greatly on adhesive bonds of carbon fiber and PTFE matrix.

The carried out research have shown that the thermal-oxidation of fiber surface is the most widespread method of carbon fiber surface modification with the purpose to improve the PTFE adhesion to CF. The thermal oxidative processing of CF surface results in an increase of specific surface and degree adsorption [28].

The peculiarities of the thermal (before milling) and thermomechanical (during milling) modification of fiber surface were studied according to the results of experiments. Table 5 contains the research results of the wear resistance of carbon fiber-reinforced plastic (CFRP) containing heat-treated fiber, which prove the effectiveness of the thermal (increase of wear resistance up to 100%) and thermomechanical modification (increase of wear resistance up to 130%) of CF.

The most effective thermal and thermomechanical modification is at 400 °C (the temperature of crystallites melting PTFE is 325–350 °C) as a result of a supermolecular structure change because of a flexibility increase of PTFE macromolecules. For the CFRP containing CF, an increase of strength by 50% and wear resistance by more than two times is observed after the thermomechanical modification.

**Figure 4:**
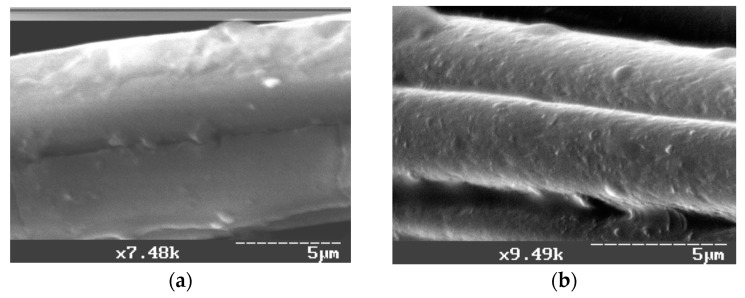
Photomicrographs of CF surfaces taken using scanning electron microscopy: (**a**) before heat treatment (×7500); and (**b**) after heat treatment (400 °C, 15 min) (×9500). Scale bar: 5 µm.

The electron microscopy data (Figure 4) signify the essential change of CF surface character after the thermal modification (Figure 4b). To intensify the energy effect during the milling of CF, the mechanical modification was carried out under vacuum (P = 550 ± 10 mm Hg). It was found that the strength of CFRP containing fiber modified under vacuum increases by almost 50% and wear resistance by 100% while milling CF under vacuum (Table 6).

Modern material science widely uses a synergetic approach to the issue of increasing composites strength, thus, this research combined the influence of thermomechanical and vacuum processing of CF on physicomechanical and tribotechnical properties of CFRP (Table 7).

As shown in Table 7, the strength of CFRP increases by more than 75% and the wear resistance increases by more than three times compared to a sample that was not subjected to thermomechanical modification of fibers at 400 °C for 15 min under vacuum (P = 550 ± 10 mm Hg).

Thus, having analyzed the series of experiments, revealing the effectiveness of the influence of different technological methods on composite properties, it turned out that the thermal vacuum technology of CF modification was the most effective as it allowed increasing the strength of PTFE composite by 18–22% and its wear resistance by 20–25%; all these present a practical interest for industrial implementation.

## 7. Peculiarities of mixing Technology of Composition Ingredients 

It is difficult to obtain a strong bond between PTFE matrix and CF using traditional methods of filler preparation. The CF surface has an insufficient effect of polytetrafluoroethylene wetting; moreover, its surface is hydrophilic which prevents the physical and chemical sorption of macromolecules of the PTFE matrix. 

The surface processing of carbon fibers promotes their interaction with composite matrix [28,57,58]. When a traditional method of obtaining carbon fiber filler [59] with length distribution according to certain dependences is used, the carbon cloth is milled in a hammer mill and cloth fibers separated by sieving are subjected to milling using the mill with bottom knives. The parameters of milling technological process guarantee a fiber ensemble of certain length distribution. However, CFs of fractional composition less than a “critical” length do not practically mix with PTFE, creating dust containing an agglomerate in the composition volume and making longer, reactive fibers greasy, and decreasing their compatibility with the PTFE matrix. It results in obtaining polymer composites with insufficiently high strength and wear resistance indices, which is conditioned by the sufficient structure heterogeneity of the created composite.

The perfection of the method for obtaining carbon filler is enabled due to the successive milling of CF in the presence of PTFE powder, allowing binding the fiber with the length less than “critical” in the CFRP agglomerate and preventing the “agglutination” with longer fibers. Such qualitative and chemically reactive carbon-fiber filler increases the strength and wear resistance of PTFE composite [32].

**Figure 5:**
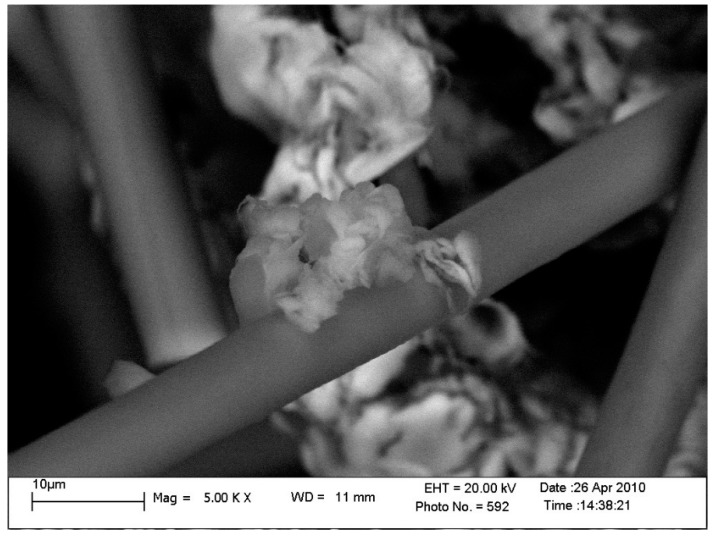
A layer with an increased content of PTFE on the surface of CF. Scale bar: 10 µm.

The structure of CF, prepared in situ, is studied with the use of scanning electron microscopy. As the photomicrography shows, there is a layer with an increased content of PTFE on the surface of CF (Figure 5).

The research analysis of experiments of the composition with CF, prepared according to the abovementioned scientific and technical solution [29], and known technological process [59], shows that the PTFE-based compositions obtained with the use of such filler increase the breaking strength by 10–20% and the wear resistance by 17–40%.

The effect of increases of composite operational properties due to the developed scientific–technological method consists in the mechanical combining of small (powdery) CF with PFTE powder which results in the creation of disperse composite product before the creation of composition in general. The composition will consist of three fractals at the end of the volume structured skeleton formation. The first fractal is flour particles of CF bonded with PFTE powder, the second is longer CF coated with PFTE (Figure 5), and the third is an unbound mass of the PTFE matrix. According to percolation theory [61], such structure of polymer composite is a prerequisite for the creation of an infinite cluster of filler (CF) in the polymer matrix (PTFE). Thus, the thermodynamic bond of small particles of fiber with necessary and sufficient volumes of PTFE powder results in obtaining more reactive filler, which has higher thermodynamic compatibility in comparison with the mechanical mixture of components in analogous proportions. This is confirmed by the data in Table 8. The introduction of such prepared CF into PTFE composition sufficiently strengthens it and increases wear resistance [37].

To increase the effectiveness of mixing PTFE with milled CF, the influence of regime staging of mixing CFRP components was studied (Table 9). The two-stage process is recommended. The milling of CF with a mixture proportion (mass) 1:1 is carried out in situ in the first stage; and the necessary additional quantity of PTFE is introduced in the second stage (optimal proportion is 1:4) [31].

As the data from Table 9 show, the strength properties of carbon fiber-reinforced plastic increase by 45% and the wear resistance increases by 80%, while producing the composition using the two-staged regime in comparison with the verification regime.

The positive effect is provided by “bonding” CF flour particles (2–60 µm) with PTFE and the creation of discrete power active centers of such a composition with high physicochemical activity as a result of the mechanical activation.

Due to high strength and wear resistance indices, the polymer composition can be used for the production of frictional unit details of energy, chemical and specialized equipment.

## 8. Technology Peculiarities of Composition Molding

One of the main drawbacks of PCM on the basis of PTFE and CF remains moisture absorption in the operation of chemical and oil equipment with liquid and gaseous media [59].

The details of the material, operating under such conditions, show an intermittent growth of wear, sometimes even critical over time. The positive results such as reducing the moisture absorption can be reached due to the optimization of the composition molding technology.

The provision of stable bond between fillers and PTFE matrix in a composite is reached due to the optimal structuring of PCM during molding. This index reflects the provision of necessary thermodynamic, kinetic, and mechanical compatibility of system ingredients, gaining maximal physical and mechanical interactions in the polymer–filler interface and the homogeneity in the macrovolume of composites, the minimization of structure defects and moisture absorption of composites while operating in the conditions of high humidity [39].

The operation characteristics and operation life are reduced significantly (3–4 times) and the wear rate rapidly increases (4–8 times) with the increase of moisture exposure on the compressor sealing composite material. It requires shutdown and unscheduled repair, which finally results in additional expenses (replacement components, equipment stoppage expenses, etc.).

As a control sample, F4CF20 (model of CFRP [16]) composite, obtained according to the known technology, with the following composition (percent (mass)) was used: PTFE, 80%; and CF, 20%, [31]. The industrial process of molding is realized under the following molding conditions: 40–45 MPa, molding speed, 0.5 × 10^−2^ m/s; and time of molding at max pressure, 300 s.

Research of the mechanism and nature of moisture absorption by CFRP, and the evaluation of property loss of the composition were carried out.

**Figure 6:**
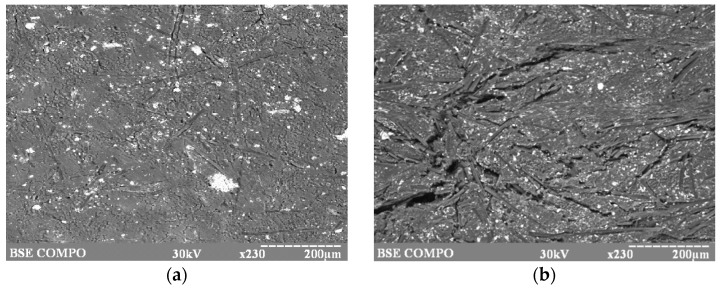
Electron photomicrographs of CFRP F4CF20 before (**a**) and after (**b**) exposition to water (×230).

The research results showed (Figure 6) that surface microdefects (Figure 6a) could appear in CFRP during product manufacturing, which can develop into destroying cracks (Figure 6b).

According to the analysis, it was found out that the moisture absorption of the composite with CF must be adjusted by the molding technology, which defines the structure, properties and the durability of composite material.

The main routes of composite moisture absorption and thus preservation of its operational properties using the obtained technology were blocked. This was accomplished by selecting optimal molding conditions; the previous processing of CF with PTFE particles; and the introduction of processing aids into CFRP.

It was estimated that the main technological characteristics that influence the components compatibility, structure and properties of composite during its molding are: pressure, molding speed, and time of molding under the pressure. These factors were taken as the main ones for experiment planning and the development of mathematical model of molding.

To gain optimal functional characteristics of PTFE composite, the crucial and main operation properties are wear rate and strength boundary at pressure, which regulate the operating capacity of frictional units in compression machines.

**Figure 7:**
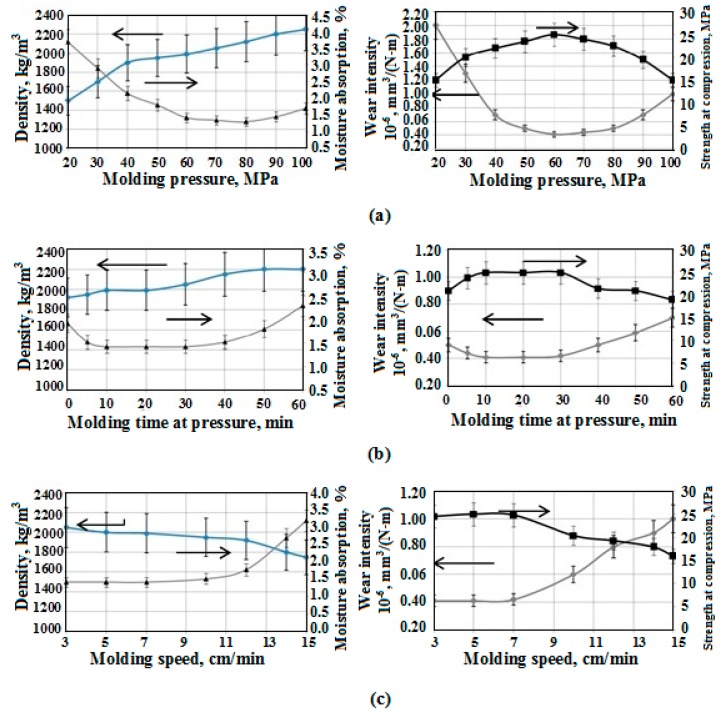
Dependences of CFRP properties on: molding pressure (**a**); time of molding under maximum pressure (**b**); and molding speed (**c**).

The research results of changes in physicomechanical and operation properties of composites depending on the technological characteristics of molding are shown in Figure 7.

**Figure 8:**
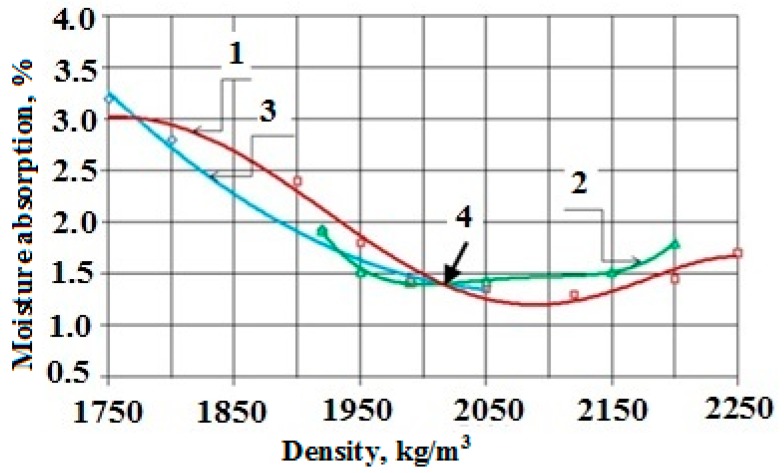
Dependence of moisture absorption on density for a variation of parameters of pressing a composite: 1, molding pressure; 2, time of molding under the pressure; 3, molding speed; and 4, optimum parameters.

It was found, and proven by the research results shown in Figure 8, that the crucial factor that sufficiently influences the moisture absorption is the composite density. In this case, the molding pressure and molding speed are the crucial technological factors.

**Figure 9:**
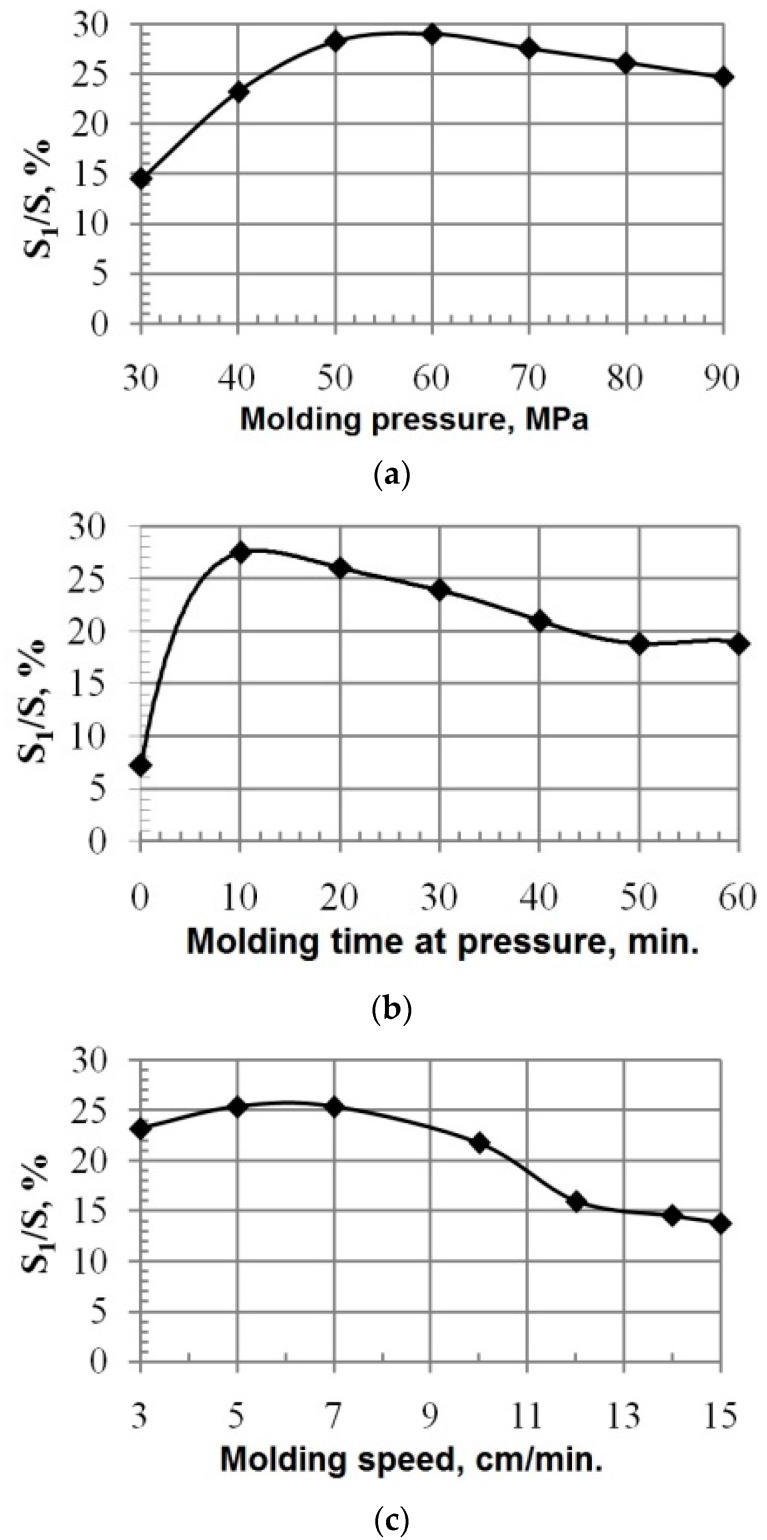
Adhesive durability of CFRP depending on technological modes of pressing process (*S*_1_—surface area of ruptures; *S*—nominal area of sample): (**a**) molding pressure; (**b**) molding time at pressure; and (**c**) molding speed.

It was theoretically grounded and experimentally proven (Figure 9) that the optimization of parameters of the PCM molding technology contributes to an increase in adhesive activity of PTFE matrix with CF.

It is conditioned by the interaction activation of radicals on interphase and, as a result, the moisture absorption is decreased and the physicomechanical, tribotechnical properties of PCM are increased.

Thus, the carried out research revealed and scientifically proved that the optimal conditions, providing stable phases interactions between PTFE matrix and CF during molding, and sufficient physicomechanical and tribotechnical properties, are the following:

- Molding pressure, 60 MPa;
- Molding speed, 0.83 m/s; and
- Molding time at pressure, 600 s.

Such conditions provide CFRP with minimal moisture absorption (less than 15–20% in comparison with the analog) and a high index of operation properties (compression strength is 15–25% higher and wear resistance is 40–45% higher than that of the analog) due to the optimal structuring of CFRP during molding.

## 9. Peculiarities of Composition Sintering Technologies

The service life of composite details depends on the heat exposure during their manufacture. The lack of sufficient information and summarized data about the impact of heat exposure on the properties of polymeric materials complicates the choice of optimal modes of the production and heat treatment of PTFE composites [61].

The technology of PTFE composites thermal processing is a thermal process of impact on the material that starts with the drying of the initial material and ends with the cooling of the heat-treated product.

The main type of thermal effect on PTFE and its composition is sintering, which consists in heating of half-finished product up to the temperature of 360–380 °C, exposure at this temperature (1 h for 1 mm thickness), and rapid cooling at temperature range from 327 °C to 350 °C.

The exposure time of the material during quenching has a significant effect on the final products’ properties. This is due to the change in the polymer structure of macromolecules configurations and the increase of straight sections numbers which become PTFE cooling crystallization centers.

Thus, the technological modes of heat processing define the degree of crystallization and, as a result, they define the physicomechanical properties of the material. The degree of crystallinity of sintered PTFE ranges 50–70% and depends on the molecular weight and the speed of cooling. The majority of PTFE mechanical properties worsen with the increase in the degree of crystallinity [61].

The research is based on the task to increase the breaking strength and wear resistance of PTFE-based composite material, reinforced with CF, due to varying sintering modes.

**Figure 10:**
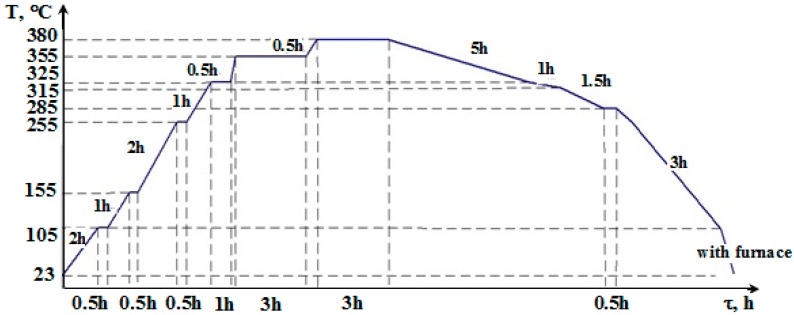
Graph of cascade heat treatment during sintering PTFE composite.

The assigned task is fulfilled because composite material sintering is carried out in the cascade heat treatment mode, taking into consideration the time of phase transition and critical points of such transition of the composite material (Figure 10).

During the sintering of billet material at the stage of the particles, fusion of polymer and carbon fiber filler, the chemical, physicomechanical, physicochemical, and electrical properties of the future product are formed in the composite. After applying the cascade heat treatment mode to the composite during sintering, which includes heating at different speeds and different time exposures with slow cooling, the shift of alternate disordered orientation and the structure stabilization at the molecular level take place. At the same time, molecule fragments orientation takes place that allow forming a more homogeneous supermolecular structure and stable composite properties in the whole volume. As a result, the strength characteristics and wear resistance of the composite are increased.

The cascade mode of sintering with a one-hour exposure at each step of cascade allows removing the uncompensated tension of molding and provides complete structural transformations in the composite volume during the transition through characteristic temperature points of phase transformations, which prevents damaging the composite integrity during cooling (cracking) and the formation of necessary correlation of polymer matrix phases.

All these allow forming the structure of CFRP of high homogeneity and stable properties of the composite. As a result, the strength and wear resistance characteristics of the composite are increased (by 25% and 50%, respectively) and it can be recommended for the production of construction materials of general and antifriction designation able to operate at high temperatures and also in chemically active media.

## 10. Conclusions

The experience of many theoretical and practical works concerning the technology of PTFE composites formation, accumulated by many prominent specialists and authors in this sphere has been analyzed and systematized. The results of this work were published in many scientific editions, but the continuation of the search for the optimal technology of PTFE composite creation presents scientific and practical interest.

The research results defined the operation regimes of process equipment: after the processing, the PTFE composites have increased operation properties in comparison with basic ones and can be most effectively used for industrial implementation:

- Energy impact on the uncompounded PTFE results in an increase in parameters of deformation and strength characteristics (breaking strength by 2.6 times, and breaking elongation by 4.3 times) while preserving high tribotechnical characteristics.
- The thermal and vacuum technology of CF modification, allowing the increase of PTFE composite strength by 18–22% and its wear resistance by 80%, was the most effective.
- By manufacturing the PTFE composition with CF using the two-stage mode, the level of strength characteristics of carbon-filled plastic in comparison with the control sample increases by 45% and wear resistance increases by 80%.
- Optimum molding modes were found, which provide stable phase interaction between the PTFE matrix and CF with minimal moisture absorption (less than 15–25% in comparison with the analog) and high operation properties (compression strength is higher by 15–25%, and wear resistance is higher by 40–45%).
- The cascade mode of sintering the PTFE composite allowed obtaining PCM, which is characterized by an increased strength and wear resistance (25% and 50%, respectively) in comparison with the analog.

Thus, PTFE composites are a bright illustration of material science triad “composition–structure–properties”. Although the processes of strengthening such materials cannot be described with the use of the universal theory, the technology of the PTFE composites of tribotechnical designation has taken place.

Modified PTFE is a further development of PTFE with improved characteristics compared with standard PTFE because of chemical modification (incorporation of a modifier in the polymer chain).

## Figures and Tables

**Table 1:** Characteristics of polytetrafluoroethylene (PTFE)—unfilled.

Characteristic	Value of the Index for the Material
Mechanical Properties	
Density, g/cc	2.13–2.19
Tensile strength (Molding direction), MPa	15–35
Elongation at break (Molding Direction), %	150–350
Hardness (Shore D)	57–64
Deformation under load, %	
1 h, 23 °C, 14.2 MPa	11.8
24 h, 23 °C, 14.2 MPa	14.3
permanent deformation	7.9
1 h, 150 °C, 5 MPa	10.0
Compressive modulus at 0.2% off-set, 23 °C, MPa	600–700
Flexural modulus at 0.2% off-set, 23 °C, MPa	690
Electrical properties	
Volume resistivity, Ω cm	10^18^
Surface resistivity, Ω	10^17^
Dielectric constant	
60 Hz	2.1
10^6^ Hz	2.1
Dissipation factor	
60 Hz	<0.0003
10^18^ Hz	<0.0003
Dielectric strength, Kv/mm	
–in air (tape)	60–80
–in oil (extruded or molded)	35–24
Thermal properties	
Melting temperature (DSC * point of fusion), °C	327
Coefficient of thermal expansion (TMA method) (23–200 °C)	
–in mold direction (MD) (10/6) °C (43 °F), ppm/°C	142
Thermal conductivity, W/(m K)	
–in molding direction	0.24
Max. working temperature, °C	260
Max. working temperature—short term, °C	300
Min. working temperature, °C	−200
Flash point, °C	530
Limiting oxygen index, %	>95
Friction Properties	
Coefficient of friction, dry sliding	
–static	0.08
–dynamic	0.06

* Differential scanning calorimetry.

**Table 2:** Chemical composition of UTM-8-1s (product model [16]) carbon fabric.

С, %	Н, %	О, %	В, %	Р, %	Ash, %
60–65	1.1–4.5	3.5–4.5	3.0–3.6	3.0–3.6	21–26

**Table 3:** Mechanical properties of UTM-8-1s carbon fabric.

Density ρ, g/cm^3^	Breaking Strength in Warp, N/cm	Breaking Strength in Transverse Direction, N/cm	Breaking Strength, GPa	Elastic Modulus, GPa	Coefficient of Thermal Conductivity, W/(m·K)
1.510	70–235	20–100	0.52–0.60	27–47	0.08–0.12

The diameter of the fibers is 10–12 µm.

**Table 4:** Effect of PTFE mechanical activation on physical and mechanical and tribotechnical properties.

No. of Sample	Technology of Obtaining	Density ρ, g/cm^3^	Breaking Strength σ_b_, MPa	Relative Elongation δ, %	Wear IntensityI∙10^−7^, mm³/N∙m
1	non-activated	2.269	9.5	96	113.3
2	τ = 3 min, *n* = 5000 min^−1^	2.208	10.2	240	108.0
3	τ = 5 min, *n* = 5000 min^−1^	2.211	21.6	416	93.0
4	τ = 8 min, *n* = 5000 min^−1^	2.175	17.3	280	80.0
5	τ = 3 min, *n* = 7000 min^−1^	2.199	10.7	270	97.0
6	τ = 5 min, *n* = 7000 min^−1^	2.205	23.5	423	82.0
7	τ = 8 min, *n* = 7000 min^−1^	2.211	18.2	358	71.7
8	τ = 3 min, *n* = 9000 min^−1^	2.203	19.6	290	89.0
9	τ = 5 min, *n* = 9000 min^−1^	2.214	24.8	415	61.0
10	τ = 8 min, *n* = 9000 min^−1^	2.213	18.0	340	72.0

**Table 5:** Wear intensity of carbon diber-reinforced plastic (CFRP; I∙10^−7^ mm^3^/N∙m) at introduction of CF after thermal and thermomechanical modification.

Modification	Control	Temperature, °C					
		100	200	300	400	500	600
Thermal	12.5	12.0	10.0	8.0	6.0	12.5	13.0
Thermomechanical		11.0	9.0	7.0	5.5	11.5	-

**Table 6:** Physical and mechanical properties of CFRP depending on conditions of grinding fiber (grinding time 15 min, vacuum P= 550 ± 10 mm Hg).

Parameter	Control	Rotation Speed of Working, min^−1^			
		7000		9000	
		Environment			
		Air	Vacuum	Air	Vacuum
Tensile strength, MPa	15.0	20.0	22.0	21.0	22.5
Wear intensity I∙10^−7^ mm^3^/N∙m	12.5	7.0	6.0	7.1	6.5

**Table 7:** Properties of CFRP at thermomechanical modification of grinding fiber on vacuum conditions (P = 550 ± 10 mm Hg).

Parameter	Control	Temperature Processing, °C				
		100	200	300	400	500
Tensile strength, MPa	15.0	24.8	24.9	25.2	26.0	24.4
Wear intensity I∙10^−7^, mm^3^/N∙m	12.5	4.4	4.4	4.2	4.0	4.5

**Table 8:** Physical, mechanical and tribotechnical properties of CFRP obtained by traditional and author’s technologies.

Parameters	Traditional Technology [35]	Author’s Technology [36]
Wear intensity I∙10^−7^, mm^3^/N∙m	8.2	4.9
Breaking strength σ_b_, MPa	18.0	24.0
Ratio of CF and PTFE powder (volume) in filler preparation	-	1:1

**Table 9:** Composition and properties of CFRP in one- and two-stage mixing modes.

Parameter	Control	Mode		
		One-Stage Mixing	Two-Stage Mixing	
			I- Mixing Stage *	II- Mixing Stage
Composition (mass %)				
PTFE	100.0	80.0	20.0	60.0
CF	-	20.0	20.0	-
Mixture after I stage	-	-	-	40.0
Properties of CFRP				
Tensile strength, MPa	15.0	20.0	-	22.0
Strength under compression, MPa	28.0	31.0	-	35.0
Relative elongation, %	20.0	20.0	-	45.0
Wear intensity I∙10^−7^, mm^3^/N∙m	12.5	9.0	-	7.0
